# Knowledge of HPV and its association with oropharyngeal cancer among dental students: a systematic review and meta-analysis

**DOI:** 10.3389/froh.2025.1604925

**Published:** 2025-05-22

**Authors:** Khaled Albusairi, Badriyah Mandani, Ward Bouresly, Yash Brahmbhatt, Hend Alqaderi, Hesham Alhazmi

**Affiliations:** 1 Kuwait Ministry of Health, Kuwait City, Kuwait; 2 Tufts University School of Dental Medicine, Boston, MA, United States; 3 Department of Public Health, Tufts University School of Dental Medicine, Boston, MA, United States; 4 Dasman Diabetes Institute, Kuwait City, Kuwait; 5 Division of Pediatric Dentistry, Department of Preventive Dentistry, Faculty of Dentistry, Umm Al-Qura University, Mecca, Saudi Arabia; 6 Department of Oral Health Policy and Epidemiology, Harvard School of Dental Medicine, Boston, MA, United States

**Keywords:** human papillomavirus, oropharyngeal cancer, dental students, knowledge assessment, HPV-related cancers, HPV awareness, dental education

## Abstract

**Background:**

Human papillomavirus (HPV) infection is a significant risk factor for oropharyngeal cancer (OPC), yet dental students' knowledge of this association varies widely. Given the critical role dentists play in early detection and prevention, understanding their level of knowledge is essential. This study systematically reviews existing research to assess dental students' awareness of HPV and its link to OPC.

**Methods:**

A systematic review and meta-analysis were conducted following PRISMA guidelines. PubMed, ProQuest, and Web of Science databases were searched for studies published up to August 2023. The Newcastle-Ottawa Scale was used to evaluate study quality. A random effects model was applied to calculate pooled prevalence with 95% confidence intervals.

**Results:**

Sixteen studies, comprising 6,345 participants, were included. The pooled analysis showed that 69% of dental students had general knowledge of HPV (range: 56%–96.5%; 95% CI: 0.56–0.81), while 77% recognized its association with OPC (range: 18%–96.4%; 95% CI: 0.63–0.89). Significant heterogeneity was observed across studies (*Q* = 646.34, *P* < 0.001 for HPV; *Q* = 804.07, *P* < 0.001 for HPV-OPC).

**Conclusion:**

Knowledge gaps among dental students may hinder prevention efforts. Standardized education in dental curricula is crucial to ensure future dentists are well-prepared to address HPV-related conditions and promote early detection in clinical practice.

## 1 Introduction

The World Health Organization (WHO) reported that cancer ranks as the second leading cause of global mortality (1). Various risk factors for oral cancer have been suggested, involving a complex interplay of factors, including the use of betel or areca nut, tobacco, and alcohol consumption (2). Furthermore, human papillomavirus (HPV) emerges as a significant risk factor for oral cancer (3). HPV is known for its sexual transmission and can infect various body regions (4). With over 40 subtypes, high-risk variants such as HPV-16 and HPV-18 are implicated in cervical, anogenital tract, and oropharyngeal cancers (OPC) (4). Approximately 4.5% of worldwide cancer cases are attributed to HPV-related OPC, giving rise to around 630,000 new cases each year (5).

In 2023, the American Dental Association recommended that dentists routinely conduct oral and oropharyngeal cancer screenings on all patients (6). These recommendations emphasize the critical role that dentists play in the early detection of oral cancers during regular check-ups to improve treatment outcomes. Given that HPV is a risk factor for oropharyngeal cancer (3), it is imperative for dentists to have a thorough understanding of different aspects of HPV and related preventive measures like early detection and vaccination. This awareness is essential not only for the early detection of oral cancers but also for highlighting the connection between oral health and overall health, positioning dentists as key players in early detection of HPV and promoting preventive healthcare measures.

Importantly, the implementation of HPV vaccines is recommended as a strategy to reduce the incidence of HPV, given their proven efficacy against cervical and anogenital tract cancers, along with their potential to mitigate oropharyngeal cancers (4). Dentists are well-positioned to educate patients about HPV transmission risks, enhance awareness, facilitate lifestyle adjustments, and actively champion participation in HPV vaccination campaigns with the overarching aim of mitigating the escalating prevalence of HPV-related oropharyngeal cancers. A deficiency of knowledge among dentists may pose a challenge to effectively delivering preventive measures to their patients.

Previous cross-sectional studies spanning various countries have investigated the knowledge of dental students regarding HPV. In the United States, Rutkoski et al. conducted a study titled “A Multi-State Evaluation of Oral Health Students” Knowledge of Human Papillomavirus Related Oropharyngeal Cancer and HPV Vaccination” (3). The survey revealed that only 18% of participants correctly identified that HPV could cause OPC (3). In Romania, Murariu et al. explored the knowledge, practice, and awareness of oral cancer and HPV infection among dental students and residents (1). One of the survey questions focused on the transmission of HPV, with 42.8% providing the correct response (1). Additionally, a 2015 study in India by Doshi et al. assessed HPV-related knowledge among female dental students, finding that 32.19% correctly associated HPV with oral cancer (7).

The most recent systematic review conducted by Kazeminejad et al. in 2021 reported that over 80% of dental students were aware that HPV can cause oropharyngeal cancer (OPC), and more than 75% of dentists acknowledged this link (8). However, no meta-analysis to date has synthesized findings on this topic. To address this gap, the present study aimed to systematically assess the level of knowledge among dental students regarding the association between HPV and OPC, and to provide informed recommendations for incorporating HPV education into dental curricula. Guided by the PICOS framework, this review included studies involving dental students that assessed educational exposure or awareness of HPV and its link to OPC, without requiring a comparator due to its prevalence-focused nature. The primary outcome was the reported level of knowledge or awareness of HPV-related OPC, which shaped the research question and eligibility criteria.

## 2 Materials and methods

### 2.1 Protocol

The systematic review and meta-analysis were conducted in accordance with the Preferred Reporting Items for Systematic Reviews and Meta-Analyses (PRISMA) guidelines. Eligible studies were selected for further investigation based on these criteria. The assessment of the level of knowledge regarding HPV and its association with OPC guided the systematic review and meta-analysis, shaping the selection of participants, data extraction, and the overall analytical framework of the study.

### 2.2 Eligibility

The Inclusion Criteria:

- Studies published up to August 2023
- Participants: Only dental students
- Study Design: Cross-sectional studies that included questions related to general knowledge about HPV and its association with OPC

Exclusion Criteria:

- Studies that included participants other than dental students
- Study designs other than cross-sectional
- Studies with questions about HPV that did not relate to general knowledge or OPC-related knowledge

### 2.3 Quality assessment

Each included study was independently reviewed by two investigators (K.A. and W.B.). The risk of bias was assessed using an adapted version of the Newcastle-Ottawa Scale, tailored specifically for cross-sectional studies. Any disagreements were resolved through consensus (9).

### 2.4 Search strategy

Databases, including PubMed, ProQuest, and Web of Science, were systematically searched for studies published up to August 2023. The search utilized terms such as “Aware*”, OR “attitude*”, OR “knowledge*”, AND “papilloma virus”, OR “human papillomavirus”, OR “HPV*”, AND “Dent*” as seen in Table 1. Additionally, a manual search was conducted to identify relevant publications. The search was restricted to English-language studies, and results were compiled into an Excel spreadsheet. Two authors independently assessed the titles and abstracts of identified studies to determine inclusion based on predefined criteria. Any discrepancies were resolved through consultation with a third author. Selected articles underwent full-text review to extract relevant information.

**Table 1:** Systematic search strategy and study selection process.

Databases	Search strategy	Date	Results
PubMed	((((((((((((((((((aware*[Title/Abstract]) OR (attitude*[Title/Abstract])) OR (opinion*[Title/Abstract])) OR (knowledge*[Title/Abstract])) OR (belie*[Title/Abstract])) OR (percept*[Title/Abstract])) OR (view*[Title/Abstract])) OR (comment*[Title/Abstract])) OR (thought*[Title/Abstract])) OR (uptake*[Title/Abstract])) OR (understand*[Title/Abstract])) OR (concept*[Title/Abstract])) OR (comprehens*[Title/Abstract])) OR (cogni*[Title/Abstract])) OR (recogni*[Title/Abstract])) OR (think*[Title/Abstract])) AND ((((papillomaviridae[Title/Abstract]) OR (“human papilloma virus”[Title/Abstract])) OR (“human papillomavirus”[Title/Abstract])) OR (HPV*[Title/Abstract]))) AND (((((((((((((((neoplas*[Title/Abstract]) OR (paraneoplas*[Title/Abstract])) OR (preneoplas*[Title/Abstract])) OR (tumor*[Title/Abstract])) OR (cancer*[Title/Abstract])) OR (precancer*[Title/Abstract])) OR (malignan*[Title/Abstract])) OR (premalignan*[Title/Abstract])) OR (benign[Title/Abstract])) OR (carcino*[Title/Abstract])) OR (precarcino*[Title/Abstract])) OR (sarcoma*[Title/Abstract])) OR (metastas*[Title/Abstract])) OR (anaplas*[Title/Abstract])) OR (dysplas*[Title/Abstract]))) AND ((dent*[Title/Abstract]) OR (student*[Title/Abstract]))	August 11th 2023	453
ProQuest	(abstract(knowledge) OR abstract(Aware)) AND (abstract(HPV) OR abstract(human papillomavirus) OR abstract(human papilloma virus)) AND (abstract (Dental))	August 16th 2023	136
WOS*	((((((TI = (knowledge)) OR (TI = (aware*))) OR (TI = (attitude*))) AND (TI = (dent*)))) AND ((TI = (HPV)) OR ((TI = (human papillomavirus)))) OR (TI = (humanpapillomavirus)))	August 11th 2023	33

### 2.5 Data analysis

The aim of this study was to assess the knowledge of dental students regarding HPV infection and its association with oral cancer. Two forest plot graphs were constructed to present the estimated effects as prevalences with 95% confidence intervals. The analysis employed the restricted maximum likelihood method, choosing between random effects or fixed effects models based on the Q statistic to indicate study heterogeneity. Significant heterogeneity prompted verification using the random effects model. Statistical significance was set at *P* < 0.05. Data analysis was performed using STATA V. 16 statistical software.

## 3 Results

### 3.1 Literature search

**Figure 1:**
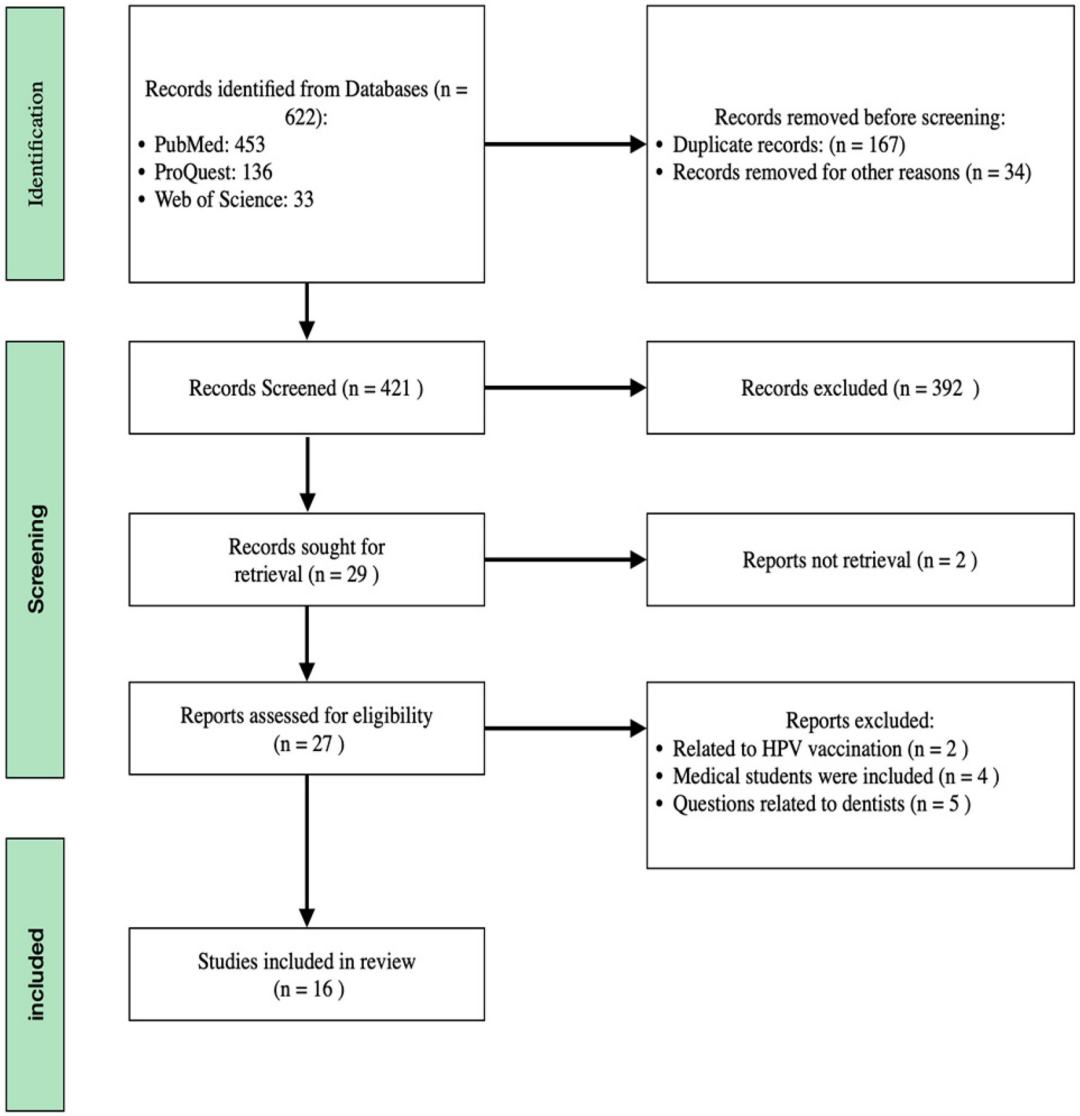
PRISMA flow chart.

**Figure 2:**
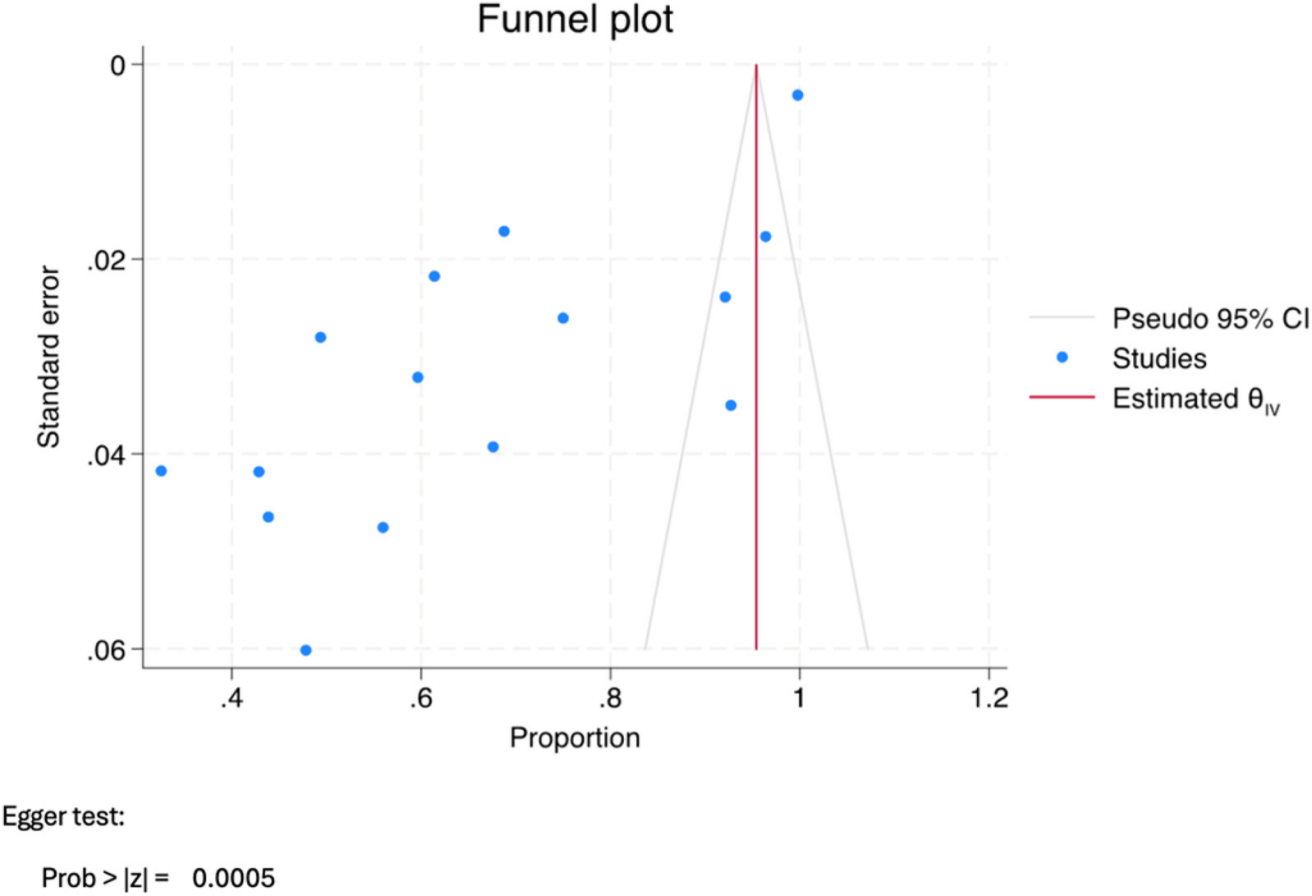
Funnel plot assessing publication bias for studies reporting general knowledge of HPV among dental students.

**Figure 3:**
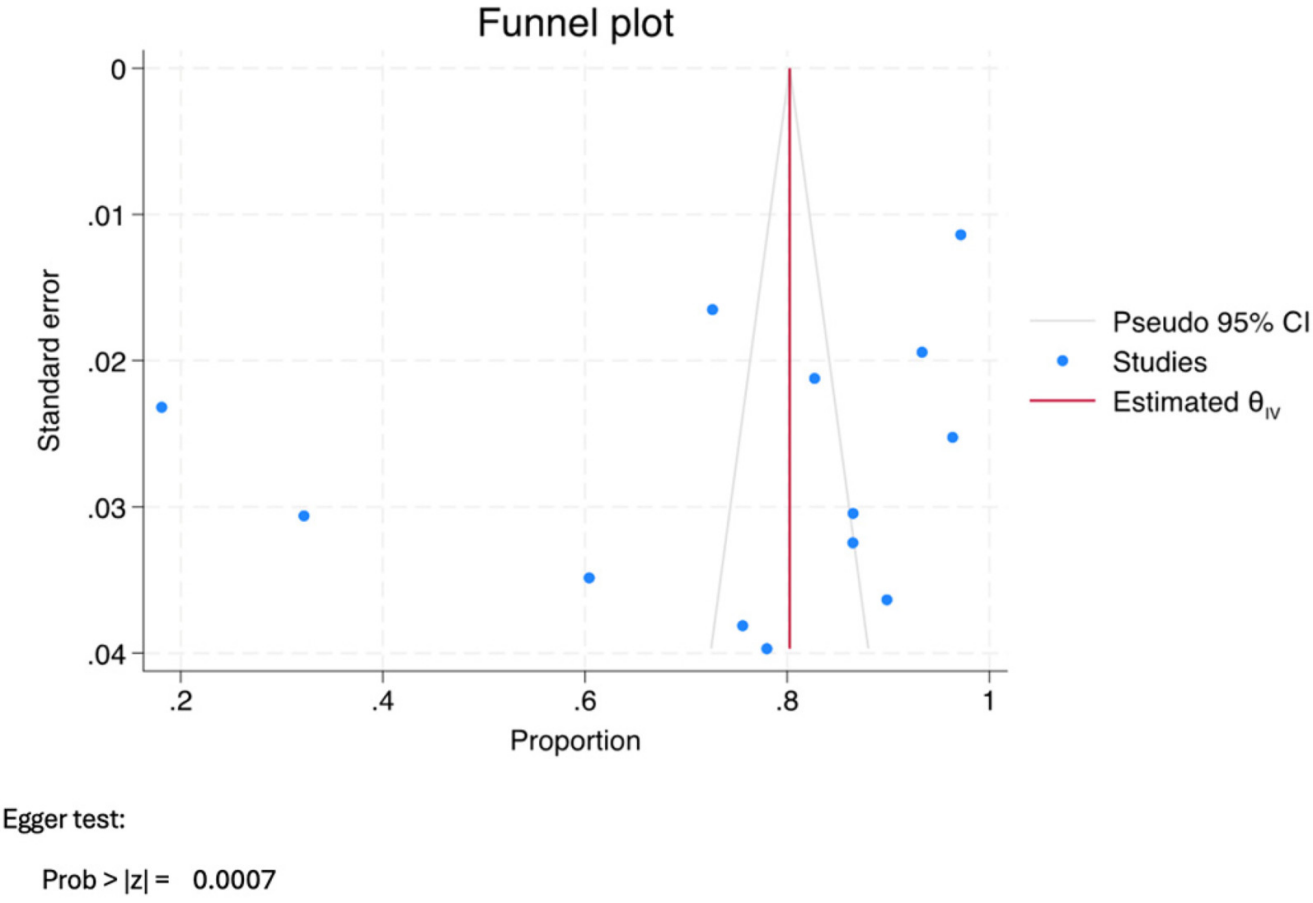
Funnel plot assessing publication bias for studies evaluating dental students’ knowledge of the association between HPV and oropharyngeal cancer (OPC).

The literature search conducted on PubMed, ProQuest, and Web of Science identified 453, 136, and 33 titles, respectively. Subsequent evaluation of these titles led to the selection of 29 relevant articles. However, upon further analysis, it was found that 11 articles did not meet the inclusion criteria, and two articles were unretrievable. Therefore, the review ultimately incorporated 16 articles. The rationale for the exclusion of specific studies and the methodology employed for article selection are visually represented in Figure 1. The category labeled “other reasons” includes exclusions due to factors such as duplicate records, irrelevance to the topic based on title and abstract screening, lack of access to full text, and article types that did not meet the inclusion criteria. Additionally, Figures 2, 3 demonstrate funnel plot analysis to assess publication bias among the included studies. Visual inspection of the plots suggested potential asymmetry in both outcomes. Egger's test confirmed statistically significant publication bias for studies reporting general HPV knowledge among dental students (*P* = 0.0005) and for those assessing knowledge of the HPV–oropharyngeal cancer (OPC) association (*P* = 0.0007). Of the 16 included studies, 15 addressed general questions regarding HPV among students, while 13 discussed the relationship between HPV and OPC.

### 3.2 Study characteristics

The characteristics of the studies included in this meta-analysis are presented in Table 2. A total of 16 cross-sectional studies were included, with 14 addressing general HPV knowledge and 13 focusing on HPV and OPC (1, 3–5, 7, 10–20). The sample sizes ranged from 55 to 730 participants, and the studies were conducted across various countries. Specifically, this systematic review included four studies from the United States conducted between 2020 and 2022, with sample sizes ranging from 55 to 276 participants. The assessment of HPV knowledge in these studies revealed percentages ranging from 56% to 96.5%. The studies also evaluated the awareness of the relationship between HPV and OPC, with awareness percentages ranging from 18% to 96.4%.

**Table 2:** Characteristics and Key findings of studies evaluating dental students*’* knowledge of human papillomavirus and oropharyngeal cancer.

Author	Country	Question related to HPV general knowledge^a^	Sample size (*n*)/Result	Question related to HPV-OPC^b^	Sample size (*n*)/Result
Torres et al. (10)	United states	HPV is a very common sexually transmitted infection, with an estimated 80% of sexually active. People contracting it at some point in their lives	*n* = 109 Result = 61 (56%)	Roughly 70% of oropharyngeal cancer is caused by high-risk HPV infections	*n* = 109 Result = 85 (78%)
Rutkoski et al. (3)	United states	General HPV knowledge question	*n* = 276 Result = 207 (75%)	HPV can cause oropharyngeal cancer	*n* = 276 Result = 50 (18%)
Wright et al. (11)	United states	There are many types of HPV (human papillomavirus)	*n* = 172 Result = 166 (96.5%)	some types of HPV are associated with approximately 70% of oropharyngeal cancers	*n* = 173 Result = 149 (86.13%)
Murariu et al. (1)	Romania	What is the method of transmission of HPV?	*n* = 140 Result = 60 (42.8%)	Is the HPV an etiological agent of oral cancer?	*n* = 197 Result = 119 (60.4%)
Chan et al. (12)	United states	HPV is a rare virus	*n* = 55 Result = 51 (92.7%)	HPV infection can cause cancers of the tongue, throat, and tonsils. (T)	*n* = 55 Result = 53 (96.4%)
Keser et al. (13)	Turkey	There are more than 100 types of HPV	*n* = 318 Result = 157 (49.3%)	Some types of HPV cause oral cancer	*n* = 318 Result = 263 (82.7%)
Doshi et al. (7)	India	HPV can affect both males and females	*n* = 233 Result = 139 (59.6%)	HPV related diseases are “oral cancer”	*n* = 233 Result = 75 (32.19%)
Rakhra et al. (14)	United Kingdom	–	–	Can HPV cause oropharyngeal cancer?	*n* = 165 Result = 154 (93%)
Sallam et al. (15)	Jordan	Have you ever heard of HPV?	*n* = 214 Result = 214 (100%)	HPV can cause oral cancer	*n* = 212 Result = 206 (97.2%)
Lingam et al. (16)	Multinational (Egypt-India-Pakistan-Saudi Arabia-UAE-Sudan)	What diseases do you know that HPV can cause?	*n* = 730 Result = 502 (68.7%)	What are the risk factors of oral cancer?	*n* = 730 Result = 530 (72%)
Lorenzo et al. (5)	Spain	There are more than 100 types of HPV	*n* = 69 Result = 33 (47.8%)	Some types of HPV cause oral cancer?	*n* = 69 Result = 62 (89.8)
Poelman et al. (17)	Netherlands	There are more than 100 types of HPV	*n* = 126 Results = 41 (32.5%)	Some types of HPV cause Oral cancer	*n* = 126 Result = 109 (86.5%)
Ozdede et al. (18)	Turkey	HPV is a bacterial infection	*n* = 127 Result = 117 (92.1%)	The same HPV types cause genital warts and OPC.	*n* = 127 Result 96 (75%)
Farsi et al. (4)	Saudi Arabia	Have you ever heard of HPV infection?	*n* = 500 Result = 307 (62%)	–	–
Pinzon et al. (19)	Latin America	There are many types of Human Papillomavirus	*n* = 114 Result = 80 (70.1%)	–	–
Rajiah et al. (20)	Malaysia	HPV infection can last for years	*n* = 142 Result = 96 (67.6%)	–	–

a HPV, human papillomavirus.

b OPC, oropharyngeal cancer.

This review also included six studies from Europe conducted between 2018 and 2022. Four of these studies assessed both HPV knowledge and the relationship between HPV and OPC, while the remaining two focused exclusively on the HPV and OPC relationship. The sample sizes for these studies ranged from 69 to 318 participants. The percentage of HPV knowledge ranged from 32.5% to 49.3%, while awareness of the HPV and OPC connection ranged from 60.4% to 93%.

Furthermore, six studies conducted between 2015 and 2022 from various countries, including those in Asia, Africa, and one in Latin America, were reviewed. Half of these studies assessed both HPV knowledge and the HPV and OPC relationship, whereas the other half focused solely on HPV knowledge. For the studies on HPV knowledge, the sample sizes ranged from 114 to 730 participants, with knowledge levels between 59.16% and 100%. For the studies addressing the HPV and OPC relationship, the sample sizes ranged from 233 to 730 participants, with awareness percentages ranging from 32.19% to 97.2%.

### 3.3 Quality assessment

Among the 16 articles included in the two meta-analyses, the quality assessment revealed that 11 articles scored 4 or less out of 9 on the NOS scale. Four studies scored 5, and only one study achieved a score of 6. The scores for each study are summarized in Table 3.

**Table 3:** Quality assessment of the studies by the Newcastle-Ottawa scale.

Author	Representative of the sample	Sample size	Non-respondents	Ascertainment of the exposure	Comparability	Assessment of outcome	Statistical test	Total
Torres et al. (10)	Zero	Zero	*	*	Zero	*	*	4
Rutkoski et al. (3)	Zero	Zero	Zero	*	Zero	*	*	3
Wright et al. (11)	Zero	Zero	*	*	Zero	*	*	4
Murariu et al. (1)	Zero	Zero	*	*	Zero	*	*	4
Chan et al. (12)	Zero	Zero	*	*	Zero	Zero	*	3
Keser et al. (13)	Zero	Zero	Zero	*	Zero	*	*	3
Doshi et al. (7)	Zero	Zero	Zero	*	Zero	*	*	3
Rakhra et al. (14)	Zero	Zero	*	*	*	*	*	5
Sallam et al. (15)	Zero	Zero	Zero	*	*	*	*	4
Lingam et al. (16)	Zero	*	*	*	Zero	*	*	5
Lorenzo et al. (5)	Zero	*	*	*	*	*	*	6
Poelman et al. (17)	Zero	Zero	Zero	*	*	*	*	4
Ozdede et al. (18)	Zero	Zero	Zero	*	Zero	*	*	3
Farsi et al. (4)	Zero	Zero	*	*	Zero	*	*	4
Pinzon et al. (19)	Zero	*	*	*	Zero	*	*	5
Rajiah et al. (20)	Zero	*	*	*	Zero	*	*	5

### 3.4 Forest plots

**Figure 4:**
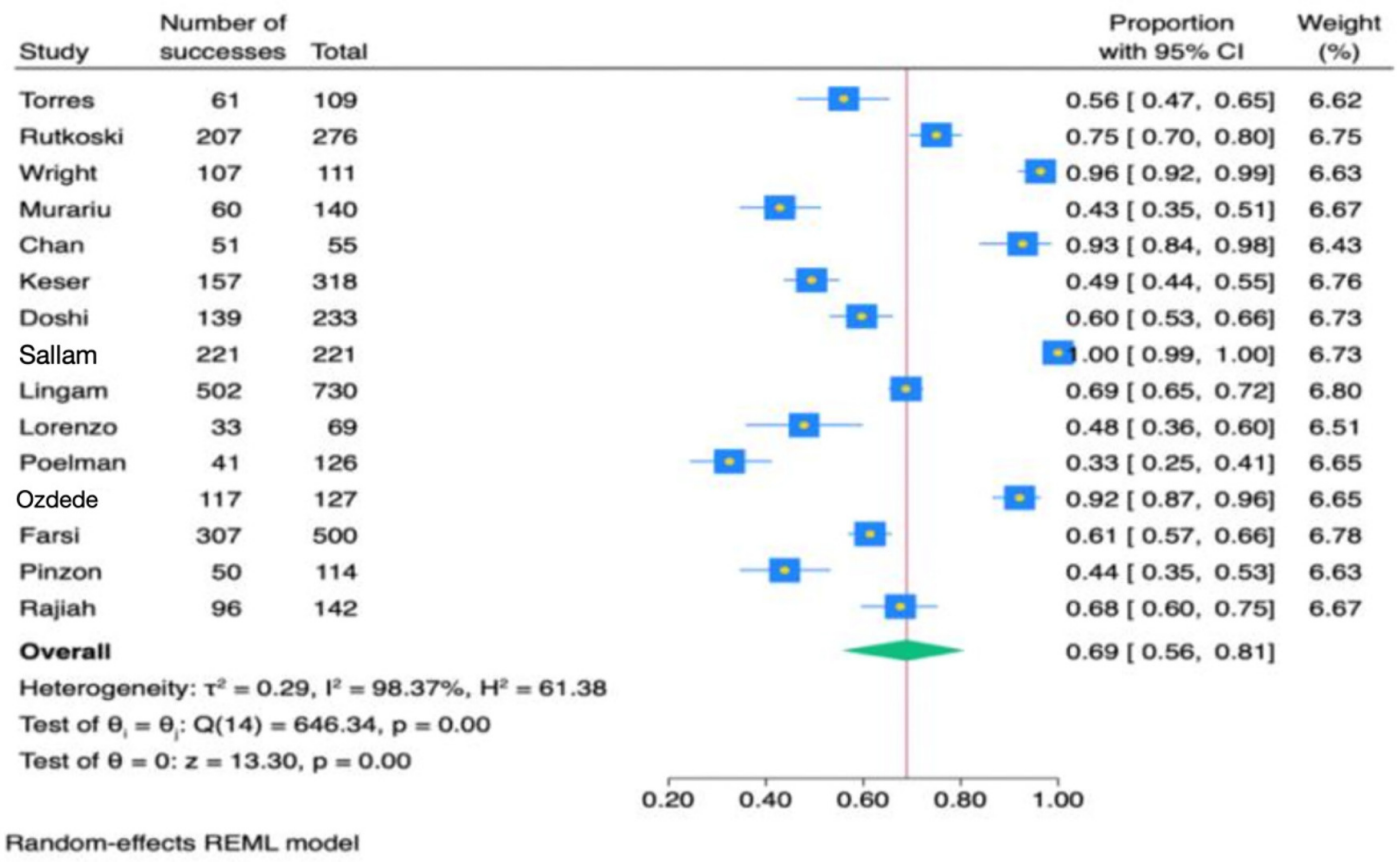
Forest plot of the estimate effect of human papillomavirus knowledge questions.

**Figure 5:**
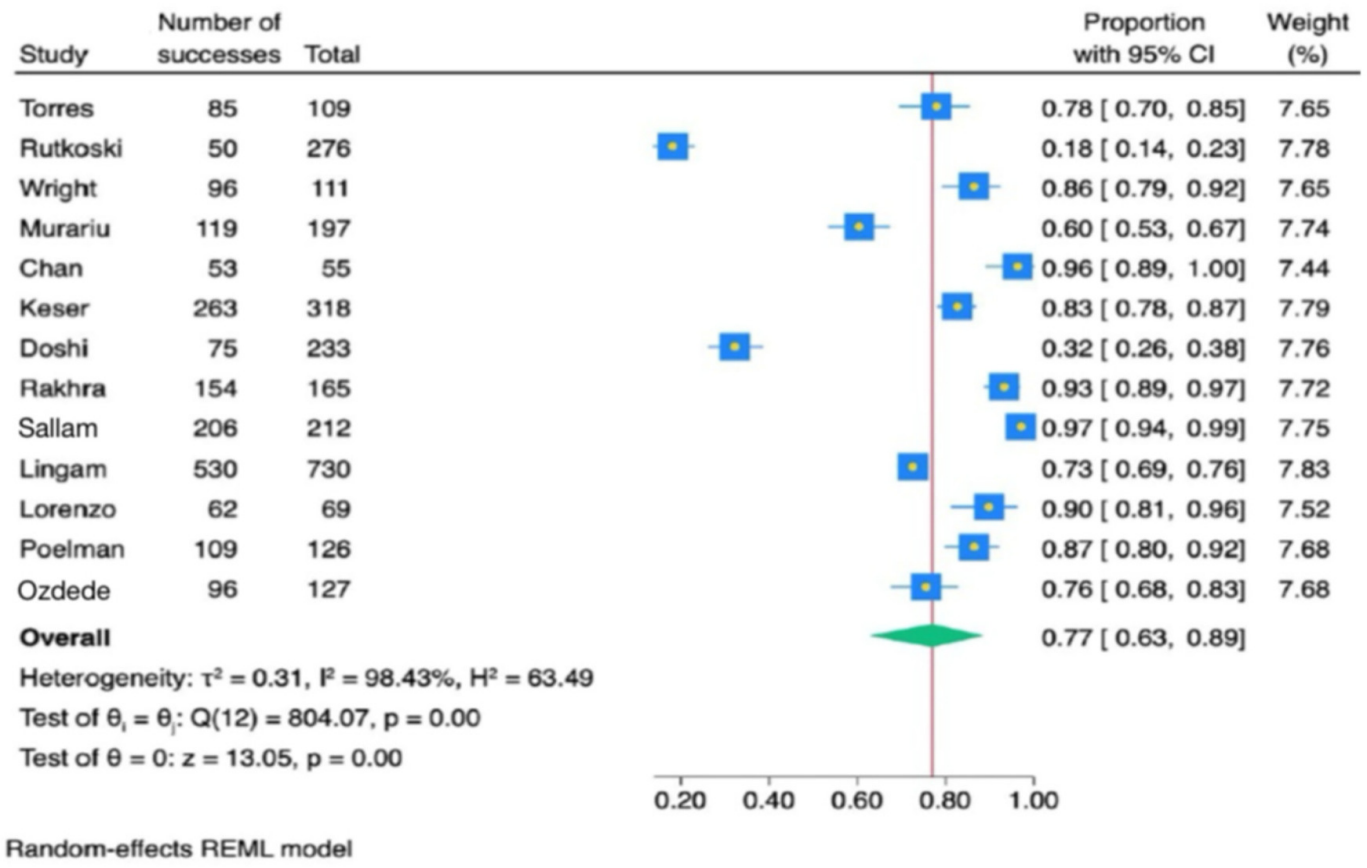
Forest plot diagram of the estimate effect of the association of human papillomavirus and oropharyngeal cancers questions.

Figure 4 presents a meta-analysis of 15 studies assessing general HPV knowledge among dental students. The findings indicate that 69% of students had general knowledge (95% CI: 0.56–0.81). Due to significant heterogeneity (*Q* = 646.34, *P* < 0.001), a random effects model was applied. Figure 5 presents a meta-analysis of 13 studies evaluating HPV-related knowledge specific to oropharyngeal cancer (OPC) among dental students. The results showed that 77% of dental students were aware of the association between HPV and OPC (95% CI: 0.63–0.89). Due to significant heterogeneity (*Q* = 804.07, *P* < 0.001), a random effects model was applied.

## 4 Discussion

This systematic review and meta-analysis study was conducted to assess the level of knowledge among dental students concerning HPV and its link to OPC. The results showed that dental students had a 69% knowledge level about HPV and a 77% understanding of HPV's association with OPC. However, the previously published systematic review which evaluated awareness of HPV-related oral cancers among both dentists and dental students, found that more than 80% of dental students are aware that HPV can cause OPC (8). Furthermore, over 75% of dentists acknowledge HPV as a causative factor in oral cancer (8). The difference between the findings in our meta-analysis and the previous systematic review might be attributed to the fact that our meta-analysis included only dental students, while the other review included both dentists and dental students. Additionally, the range of questions included in this meta-analysis varied widely. For example, 96% of the dental students chose the correct answer for the statement “There are many types of HPV”, however, knowledge on the mode of HPV transmission was lower, with only 42.8% of responses being correct.

Similarly, geographical differences and the diversity of educational systems globally might have an impact on the results observed in studies assessing dental students' knowledge about HPV and its association with OPC. For instance, in Turkey, only 49% of participants correctly answered a general question about HPV types (13). In Italy, a 47% accuracy for a similar question was reported (5). In contrast, in the United States, 96% of participants correctly identified HPV types (11). Studies conducted in the U.S. generally indicate a higher level of awareness among dental students regarding the connection between HPV and OPC (3, 11). This variation highlighted the influence of geographical location and educational frameworks on students' understanding of critical health issues, emphasizing the need for tailored educational interventions to bridge knowledge gaps worldwide.

The Commission on Dental Accreditation (CODA), which accredits dental education programs in the United States, has implemented a mandatory standard (standard 2.24-part b) (21), requiring graduates to be competent in providing oral health care within the scope of general dentistry, including screening and risk assessment for head and neck cancer (21). Thus, dental schools must develop and update their curricula to meet these national standards. These standards also have the potential to ensure comprehensive dental education that integrates HPV knowledge and enhances early detection of head and neck cancers globally. Integrating a standardized component into dental curricula is essential to ensure that all dental students gain comprehensive knowledge about HPV and learn to apply this knowledge in their clinical practice. This should include training on oral cancer screening, patient education about HPV, and the importance of vaccination. By enhancing both the theoretical and practical aspects of dental education to include thorough information on HPV and its health implications, we can better prepare dental students with the skills and expertise necessary to diagnose, understand, and manage HPV-related conditions effectively in their professional careers (3, 4, 11, 7, 13, 15).

A systematic review assessed healthcare providers' knowledge of HPV, with sample sizes ranging from 172 to 194, revealing that knowledge levels varied between 21% and 84% (22). Comparatively, dental students in this study showed an average knowledge level of 69%, ranging from 56% to 96.5%. Moreover, the review examined the understanding of the link between HPV and oropharyngeal squamous cell carcinoma, noting knowledge levels among providers ranged from 22% to 100%. In contrast, dental students demonstrated an average knowledge level of 77% on questions pertaining to HPV and OPC (3, 4, 11).

While this meta-analysis provides valuable insights into dental students' knowledge of HPV and its association with OPC, it is important to consider several limitations. Primarily, the effect estimates are derived from cross-sectional studies, which inherently carry risks of bias, including selection, information, and recall biases. These biases could compromise the accuracy and applicability of the findings (3, 4). The quality of the included studies was evaluated using the Newcastle-Ottawa Scale (NOS), which revealed low scores on quality indicators (8). Another significant limitation is the representativeness of the study samples; none of the analyzed studies included samples that were fully representative of the target population, thereby limiting the generalizability of the findings to the broader population of dental students (16). Additionally, only four out of the sixteen studies reviewed had sample sizes that were deemed adequate and satisfactory, potentially restricting the validity of the meta-analysis outcomes (19). Most studies in this meta-analysis did not control for confounding factors, which could skew the true effect of the relationship between dental students' knowledge of HPV and its implications for oral health (3, 4). Lastly, it is important to acknowledge that each study used different questions, which may have been perceived differently by participants across studies and countries. This variability in survey instruments and question wording may have contributed to the heterogeneity between studies observed in the meta-analysis. To address this, we employed a random-effects model to account for the heterogeneity.

Given these limitations, the findings of this meta-analysis should be interpreted with caution. Future research should aim to address these limitations by conducting studies with more rigorous designs, larger and more representative samples, and comprehensive controls for confounding variables to provide more reliable and generalizable results (1, 8, 14).

## 5 Conclusions

This study underscores disparities in HPV-related knowledge across dental student cohorts. While certain students exhibit a robust comprehension of HPV and its clinical ramifications, others display substantial knowledge gaps. These findings underscore the imperative to implement structured educational interventions within dental curricula. Such initiatives are essential to equip future dentists with the requisite proficiency in early detection, prevention, and management of HPV-related oral health conditions.

## Data Availability

Publicly available datasets were analyzed in this study. This data can be found here: N/A.
