# Correction: Knowledge of HPV and its association with oropharyngeal cancer among dental students: a systematic review and meta-analysis

**DOI:** 10.3389/froh.2026.1815414

**Published:** 2026-03-12

**Authors:** Khaled Albusairi, Badriyah Mandani, Ward Bouresly, Yash Brahmbhatt, Hend Alqaderi, Hesham Alhazmi

**Affiliations:** 1 Kuwait Ministry of Health, Kuwait City, Kuwait; 2 Tufts University School of Dental Medicine, Boston, MA, United States; 3 Department of Public Health, Tufts University School of Dental Medicine, Boston, MA, United States; 4 Dasman Diabetes Institute, Kuwait City, Kuwait; 5 Division of Pediatric Dentistry, Department of Preventive Dentistry, Faculty of Dentistry, Umm Al-Qura University, Mecca, Saudi Arabia; 6 Department of Oral Health Policy and Epidemiology, Harvard School of Dental Medicine, Boston, MA, United States

**Keywords:** human papillomavirus, oropharyngeal cancer, dental students, knowledge assessment, HPV-related cancers, HPV awareness, dental education

In the article, the Acknowledgements section was unintentionally omitted from the original version of this article. The Acknowledgements section should read as “The authors acknowledge The Kuwait Foundation for the Advancement of Sciences (KFAS).” The original version of this article has been updated.

